# Simultaneous Suppression of Two Distinct Serotonin *N*-Acetyltransferase Isogenes by RNA Interference Leads to Severe Decreases in Melatonin and Accelerated Seed Deterioration in Rice

**DOI:** 10.3390/biom10010141

**Published:** 2020-01-15

**Authors:** Ok Jin Hwang, Kyoungwhan Back

**Affiliations:** Department of Biotechnology, College of Agricultural and Life Sciences, Chonnam National University, Gwangju 61186, Korea; smilax@jnu.ac.kr

**Keywords:** melatonin, RNA silencing, seed viability, *SNAT1*, *SNAT2*, transgenic rice

## Abstract

Serotonin *N*-acetyltransferase (*SNAT*) is the penultimate enzyme in the melatonin biosynthetic pathway, in which serotonin is converted into *N*-acetylserotonin (*NAS*) in plants. To date, two *SNAT* isogenes with low amino acid sequence homologies have been identified. Their single suppression in rice has been reported, but their double suppression in rice has not yet been attempted. Here, we generated double-suppression transgenic rice (*snat1+2*) using the RNA interference technique. The *snat1+2* exhibited retarded seedling growths in conjunction with severe decreases in melatonin compared to wild-types and single-suppression rice plants (*snat1* or *snat2*). The laminar angle was decreased in the *snat1+2* rice compared to that of the wild-types and *snat1*, but was comparable to that of *snat2*. The reduced germination speed in the *snat1+2* was comparable to that of *snat2*. Seed-aging testing revealed that *snat1* was the most severely deteriorated, followed by *snat1+2* and *snat2*, suggesting that melatonin is positively involved in seed longevity.

## 1. Introduction

Melatonin is a multifunctional regulator, serving as a signal molecule for various metabolic processes, and is also a potent antioxidant in plants [1,2,3,4]. Melatonin influences not only key plant hormones, such as auxin and cytokinin, which are essential for plant growth and development [5], but also plant redox signaling molecules, such as reactive oxygen species (ROS) and reactive nitrogen species (RNS), which play pivotal roles in numerous processes, including seed germination, senescence, and various stress responses [6]. Due to the large number of physiological roles played by melatonin, it has long been suspected that plants, like animals, possess melatonin receptors [7]. Recently, it was reported that *Cand2* serves as a melatonin receptor involved in stomatal closure in *Arabidopsis* [8]. However, further in-depth studies are needed to understand the involvement of *Cand2* in other melatonin-mediated plant functions, such as the induction of mitogen-activated protein kinase [9].

In plants, melatonin biosynthesis consists of four enzymatic steps, beginning with the aromatic amino acid tryptophan [10]. In plants, tryptophan decarboxylase (*TDC*) catalyzes the first committed step, in which tryptophan is converted into tryptamine, whereas, in animals, tryptophan hydroxylase (*TPH*) is the first enzyme, which catalyzes tryptophan conversion into 5‑hydroxytryptophan [2]. In plants, tryptamine is further hydroxylated into serotonin by the action of tryptamine 5-hydroxylase (*T5H*), a P450 enzyme localized in the endoplasmic reticulum. The penultimate enzyme is serotonin *N*-acetyltransferase (*SNAT*), which converts serotonin into *N*-acetylserotonin. The last enzyme is an *O*-methyltransferase that catalyzes the conversion of *N*-acetylserotonin into melatonin; both *N*-acetylserotonin *O*-methyltransferase (*ASMT*) and caffeic acid *O*-methyltransferase (*COMT*) are involved. The rate of serotonin synthesis is much greater than the rate of serotonin conversion into melatonin; thus, the latter half of the pathway plays a pivotal role in regulating melatonin levels in plants [10]. *SNAT* is believed to be the rate-limiting step enzyme, rather than *ASMT*, in view of the catalytic efficiencies (*V*_max_/*K*_m_) of the respective enzymes [10]. In 2013, the first *SNAT* gene (*SNAT1*) was cloned from rice [11]. The overexpression of *SNAT1* conferred tolerance against abiotic stress [12], whereas its downregulation (*snat1*) led to a susceptibility to abiotic stress and retarded seedling growths, in conjunction with altered melatonin levels [13]. In marked contrast, downregulation of *SNAT2* in rice (*snat2*) led to an enhanced tolerance to abiotic stress, a semidwarf phenotype with erect leaves, and a decrease in melatonin content [14,15]. The major difference between *snat1* and *snat2* rice was the level of brassinosteroid (BR), of which *snat2* was more deficient than the wild-type, while *snat1* had a BR content comparable to that of the wild-type. Both *snat1* and *snat2* rice had decreased melatonin levels, but they had different phenotypes and abiotic stress responses, depending on their BR levels [15,16]. These contradictory results prompted us to generate a double-suppression rice mutant (*snat1+2*) to observe the effects of the simultaneous downregulation of both genes.

## 2. Materials and Methods

### 2.1. Generation of a Double-Suppression Rice Mutant (snat1+2) Using RNA Interference

Rice (*Oryza sativa*) has two *SNAT* isogenes: *SNAT1* and *SNAT2,* which share 27% amino acid identities [14]. *SNAT1* is located on chromosome 5, whereas *SNAT2* is located on chromosome 8 [17]. The full-length *SNAT1* (GenBank accession number AK059369) and *SNAT2* (GenBank accession number AK068156) genes from rice were provided by the National Institute of Agrobiological Sciences (NIAS, Tsukuba, Japan) [17]. To simultaneously knock down *SNAT1* and *SNAT2* gene expressions in rice, a chimeric gene composed of *SNAT1* and *SNAT2* was constructed as follows: The *SNAT1* insert, positioned in the middle of the cDNA, was PCR-amplified with forward (5′-ACT AGT ACT CCT AGA AAG-3′ [*Spe*I site underlined]) and reverse (5′-CTC GAG AGG CAT AGT AAC TGA-3′ [*Xho*I site underlined]) primers. The *SNAT2* insert, positioned in the middle of the cDNA, was PCR-amplified with forward (5′-CTC GAG GCG GGG GAC GGC GTG-3′ [*Xho*I site underlined]) and reverse (5′-GAG CTC GGG GTC CAT GGC GAA-3′ [*Sac*I site underlined]) primers. Both PCR fragments were digested by *Xho*I and ligated together (RBC Bioscience, New Taipei City, Taiwan). Using the ligated product as a template, PCR was carried out to obtain a chimeric gene (*SNAT1* + *SNAT2*) with a primer set (forward 5′-ACT AGT ACT CCT AGA AAG-3′ [*Spe*I site underlined] and reverse 5′-GAG CTC GGG GTC CAT GGC GAA-3′ [*Sac*I site underlined]). The resulting 393-bp chimeric fragment was cloned into the T&A cloning vector (T&A:OsSNAT1+2; RBC Bioscience), and the antisense *SNAT1+2* insert was obtained by *Sac*I and *Spe*I double-digestion and ligated into the pTCK303 binary vector [18], which had been digested by the *Sac*I and *Spe*I restriction enzymes. Thereafter, the sense fragment of the *SNAT1+2* insert was from *Kpn*I and *Bam*HI digestion from the T&A:OsSNAT1+2 plasmid and purified on DE81 ion exchange paper (Whatman, Maidstone, UK). The purified *Kpn*I and *Bam*HI insert was further ligated into the pTCK303 vector harboring the corresponding antisense *SNAT1+2* fragments, which were predigested with *Kpn*I and *Bam*HI.

**Figure 1:**
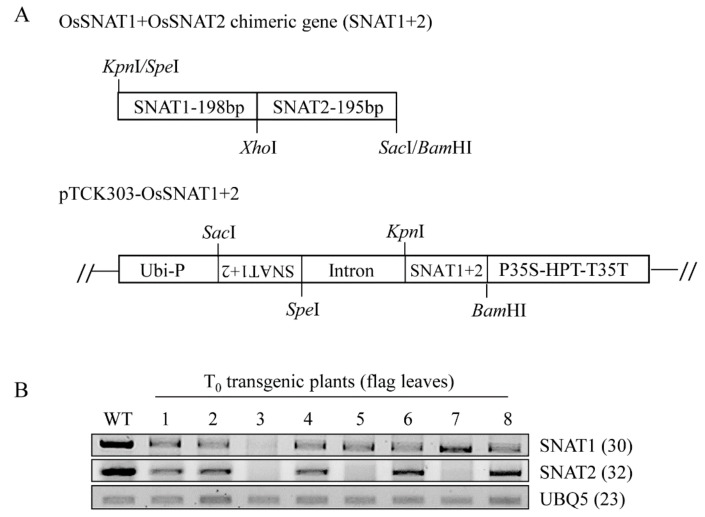
Schematic diagram of the *SNAT1+2* chimeric gene construct and reverse transcription–polymerase chain reaction (RT-PCR) analyses of T_0_ transgenic rice plants. (**A**) Construction of chimeric gene containing *SNAT1* and *SNAT2* and the binary vector used for *SNAT1+2* suppression. (**B**) RT-PCR analyses of independent T_0_ transgenic lines grown for 15 weeks in a paddy field. *SNAT* = serotonin *N*-acetyltransferase, *Ubi-P* = maize ubiquitin promoter, HPT = hygromycin phosphotransferase, WT = wild-type, *UBQ5* = rice ubiquitin5 gene, and 1–8 = *SNAT1+2*‑underexpression line. The GenBank accession numbers of *SNAT1, SNAT2,* and *UBQ5* are AK059369, AK068156, and Os03g13170.

The pTCK303:OsSNAT1+2 RNAi binary vector (Figure 1A) was transformed into *Agrobacterium tumefaciens* LBA4404 using the freeze-and-thaw method, followed by transformation into rice, as described previously [19].

### 2.2. Plant Growth Conditions

Seeds of wild-type (*Oryza sativa* cv. Dongjin) and transgenic rice were soaked in sterile distilled water for three days at 28 °C, and the germinated seeds were transplanted into soil for seed production. The plants were grown in a culture room at 28 °C/24 °C (day/night) with a 14 h-light/10 h-dark cycle or in a paddy field at the Chonnam National University (53 m a.s.l.; 35°09′ N and 126°54′ W), Gwangju, Korea. The paddy field was a controlled area for growing the transgenic rice plants permitted by the Rural Development Administration of Korea. The angles of the lamina joints of the second leaves were measured in 14-day-old rice seedlings. The RNAi lines of rice *SNAT1* (*s1*) and *SNAT2* (*s2*) were obtained from previous reports [13,14]. Fertilizer was applied at 70 N/40 P/70 K kg/ha. The rice seeds were harvested by cutting mature panicles with a scissor, and spikelets were hand-threshed.

### 2.3. Semi-Quantitative Reverse Transcription–Polymerase Chain Reaction (RT-PCR) Analysis and Quantitative Real-Time (qRT)-PCR Analyses

The total RNA of the rice plants was isolated using a NucleoSpin RNA Plant Kit (Macherey-Nagel, Düren, Germany). First-strand cDNA was synthesized from 2 μg of total RNA using MG MMLV Reverse Transcriptase (MGmed, Inc.; Seoul; Korea) and an oligo dT_18_ primer at 42 °C for 1 h. Semi-quantitative RT-PCR was performed, as described previously [14]. Real-time PCR was performed in a Mic qPCR cycler system (Biomolecular Systems, Queensland, VIC, Australia) with specific primers and the Luna Universal qPCR Master Mix (New England Biolabs, Ipswich, MA, USA). The expressions of genes were analyzed using Mic’s RQ software (Biomolecular Systems) and normalized to UBQ5. Semi-quantitative RT-PCR and real-time PCR were performed with the following primer set: SNAT1 forward 5′-CAG TAG AGC CAC CAT CAG CA-3′, SNAT1 reverse 5′-ATC CCA CCT TGT CGC ATA AA-3′, SNAT2 forward 5′-GTC TGG GAC GTG GTC GTG-3′, SNAT2 reverse 5′-GTT GCC TTG AGC GGT AGA AG-3′, DWARF4 forward 5′-GTG CTG CCA TTC TCG GAG TAA TAG-3′, DWARF4 reverse 5′-CTC AGC AAG AGG TCC AGG ATT TGC-3′, UBQ5 forward 5′-CCG ACT ACA ACA TCC AGA AGG AG-3′, and UBQ5 reverse 5′-AAC AGG AGC CTA CGC CTA AGC-3′.

### 2.4. Quantification of Melatonin

Frozen rice samples (0.1 g) were ground into powder in liquid nitrogen using the TissueLyser II (Qiagen, Tokyo, Japan) and extracted with 1 mL of chloroform for 24 h at 4 °C. The chloroform extracts (200 µL) were completely evaporated and dissolved in 0.1 mL of 40% methanol, and 20 µL aliquots were subjected to high-performance liquid chromatography using a fluorescence detector system (Waters, Milford, MA, USA). Melatonin was detected by emissions at 348 nm, using 280 nm excitation. All measurements were taken in triplicate.

### 2.5. Germination Assay

Seeds of wild-type and transgenic rice (*n* = 25) were surface-sterilized and germinated in 3 mL distilled water in six-well plates in a culture room at 28 °C/24 °C (day/night) with a 14 h‑light/10 h-dark cycle. A seed was considered to have germinated if the seed coat was ruptured and a radicle of >1 mm in length had emerged. The germination speed index (GSI) was calculated using the formula GSI = (G_1_/N_1_) + (G_2_/N_2_) + … +(G_n_/N_n_) reported by Maguire [20]. Each treatment was replicated three times.

### 2.6. Giberellic Acid (GA) Treatment for Measuring Third-Leaf Sheath Elongations

Rice seeds were surface-sterilized and placed on half-strength Murashige and Skoog (MS) mediums (MB Cell, Seoul, Korea) at 28 °C/24 °C (day/night) with a 14 h-light/10 h-dark cycle for four days and then transferred to half-strength MS mediums containing 10 μM GA_3_. The length of the third-leaf sheaths (*n* = 15) were measured four days after applications of GA_3_ [21]. All measurements were taken in triplicate.

### 2.7. Accelerated Aging Treatment and Seed Germination Determination

An accelerated aging treatment was performed, as described previously [22]. To accelerate aging, rice seeds were incubated at 42 °C and 100% relative humidity in a growth cabinet for four days. These aged seeds were then surface-sterilized and soaked in sterile distilled water. Germination tests (*n* = 25) were carried out in a culture room at 28 °C/24 °C (day/night) with a 14 h‑light/10 h-dark cycle for six days. All measurements were taken in triplicate.

### 2.8. Statistical Analysis

Data were analyzed by analysis of variance using IBM SPSS Statistics 23 software (IBM Corp. Armonk, NY, USA). Means with different letters or asterisks indicate significantly different values at *p* < 0.05, according to Tukey’s post-hoc honestly significant difference (HSD) test. Data are presented as means ± standard deviations.

## 3. Results and Discussion

### 3.1. Generation of SNAT1+2 Double-Suppression Transgenic Rice Plants

Due to a low amino acid sequence identity between rice *SNAT1* and rice *SNAT2* (>27%), there was no highly homologous DNA region common to both of them. Thus, a chimeric gene comprising *SNAT1* and *SNAT2* was constructed and used as a single-transgene construct for downregulation of both *SNAT1* and *SNAT2* genes (Figure 1A). This approach was successful in the suppression of three members of the *OsRac* gene family by a single chimeric construct [23].

**Figure 2:**
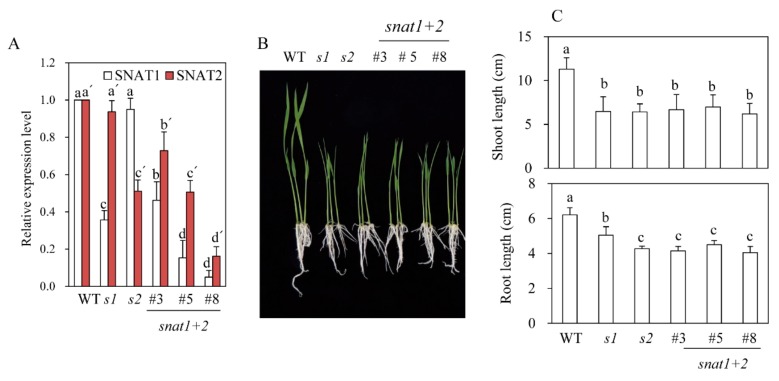
Expressions of *SNAT* isogenes and growths of *snat1+2* rice seedlings. (**A**) qRT-PCR analysis of *SNAT1* and *SNAT2* messenger RNA (mRNA) in *snat1+2* rice. (**B**) Seedling phenotypes of *snat1+2* rice grown for one week. (**C**) Shoot and root lengths of *snat1+2* (*n* = 20). *s1* = *snat1* rice, *s2* = *snat2* rice, and *snat1+2* = double-suppression rice. Different letters indicate significant differences from the wild-types (Tukey’s post-hoc honest significant difference (HSD) test; *p* <0.05).

Through the scutellum-derived calli transformation by Agrobacterium infections, we first generated eight independent T_0_ transgenic rice plants (snat1+2), in which both SNAT1 and SNAT2 genes were downregulated, as indicated by RT-PCR analyses (Figure 1B). By self-crossing T_1_ snat1+2 rice, we selected and generated three independent homozygous lines of T_2_ snat1+2 rice: lines 3, 5, and 8. Although the expression levels of both SNAT1 and SNAT2 in the T_2_ rice were diminished, compared with the wild-type, the degree of suppression was significantly greater for SNAT1 than SNAT2 when measured by qRT-PCR (Figure 2A). This result indicates that the simultaneous suppression of both SNAT1 and SNAT2 was stably transmitted from the T_0_ to T_2_ generation. The variations of SNAT1 and SNAT2 expressions between the snat1+2 lines were attributed to the positional effects, indicating that the T-DNA in the three independent homozygous lines was inserted in different loci of rice chromosomes. Collectively, we successfully generated three T_2_ homozygous snat1+2 transgenic rice lines exhibiting suppression of both SNAT1 and SNAT2 mRNA compared to the wild-types (Figure 2A). When grown in MS mediums, the snat1+2 rice exhibited retarded seedling growths and inhibited growths of both the shoots and roots (Figure 2B,C). Compared to single-suppression rice, such as snat1 (s1) and snat2 (s2), the shoot lengths of snat1+2 were comparable to those of s1 and s2, and the root lengths of snat1+2 were equal to those of s2. Based on the seedling phenotypes, the snat1+2 double-suppression rice was similar to s2.

### 3.2. Quantification of Melatonin in snat1+2 Rice

**Figure 3:**
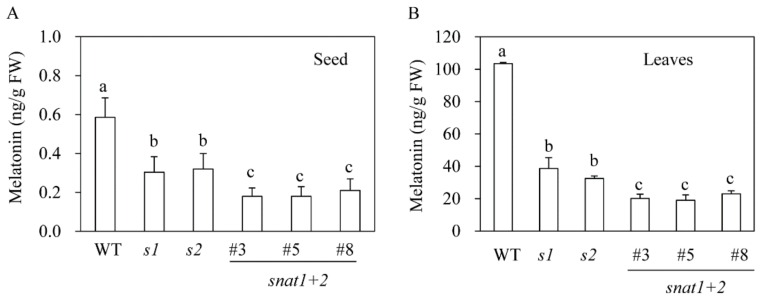
Melatonin content of seeds and leaves. (**A**) Melatonin content of seeds. (**B**) Melatonin content of rice leaves upon cadmium treatment. Seeds imbibed water for 9 h and were then subjected to high-performance liquid chromatography (HPLC) analysis for melatonin quantification. Seven-day-old rice seedlings were rhizospherically challenged with 500 μM CdCl_2_ for three days to induce melatonin production, and leaves were subjected to melatonin analysis.

To determine whether the suppression of *SNAT* was closely associated with melatonin synthesis, we first measured melatonin content in dehulled seeds that had imbibed water for 9 h. As shown in Figure 3A, wild-type seeds contained 0.6 ng/g fresh weight (FW) melatonin, whereas the *s1* and *s2* mutants contained approximately half as much melatonin. Furthermore, the *snat1+2* rice contained significantly less melatonin than either the *s1* or *s2* lines. Melatonin was also measured in seven-day-old rice seedlings that had been rhizospherically challenged with 0.5 mM cadmium chloride for three days to induce melatonin biosynthesis under continuous light. Under these conditions, the wild-types had melatonin levels of 100 ng/g FW, whereas the *s1* and *s2* lines produced 38 ng/g FW and 29 ng/g FW, respectively. Not surprisingly, the melatonin content was smallest in the *snat1+2* rice (20 ng/g FW). These data suggest that *SNAT1* and *SNAT2* are synergistically involved in melatonin synthesis in rice.

### 3.3. Leaf Angle Phenotypes

**Figure 4:**
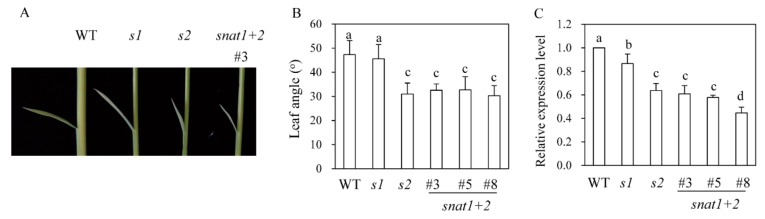
Measurements of second leaf angles and *DWARF4* expressions. (**A**) Photograph of representative transgenic rice plants. (**B**) Leaf angle measurements (*n* = 10). (**C**) Relative expression levels of *DWARF4*.

Previous research has revealed that the *s2* rice exhibits a phenotype of dwarf plants with erect leaves [14], whereas the *s1* phenotype is a dwarf without erect leaves [13,15]. The erect leaves in *s2* rice were attributed to a decrease in BR content [14]. As shown in Figure 4, the *snat1+2* rice was phenotypically similar to *s2* plants, with a decreased leaf angle, while *s1* plants were phenotypically similar to the wild-types with respect to leaf angle. *DWARF4*, a key BR biosynthetic gene, was significantly reduced in *s2* and *snat1+2,* compared to *s1* (Figure 4C), indicative of severe BR decreases in both *s2* and *snat1+2,* compared to *s1* and wild-types. *DWARF4* is a rate-limiting step enzyme that catalyzes C22-hydroxylation from 6-oxo-campestanol into cathasterone. The failure or reduction of *DWARF4* expression leads to dwarf, erect leaf, and aberrant skotomorphogenic phenotypes in plants [14,15]. These observations suggest different physiological roles for the *SNAT1* and *SNAT2* genes, although they are both involved in melatonin biosynthesis in rice plants. Leaf angle is affected by plant hormones, such as BR and GA [24]. Thus, it is likely that melatonin interacts with these plant hormones to orchestrate plant growths and developments in much the same way that melatonin interacts with key molecules in animals [2,25,26].

### 3.4. Germination Speed and GA Response to Leaf Sheath Growths

**Figure 5:**
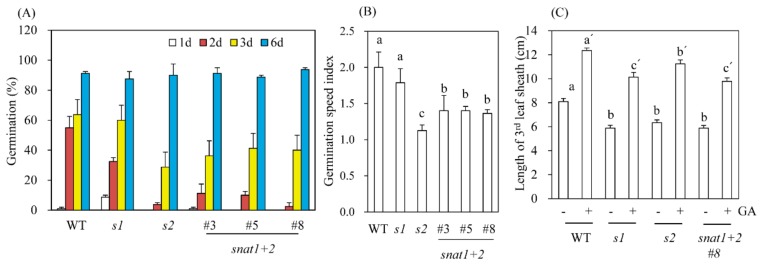
Germination rates, speeds, and increases in leaf sheaths after GA_3_ treatments. (**A**) Time–course experiment of germination rates. (**B**) Germination speed index. (**C**) Length of third-leaf sheaths in response to 10 μM GA_3_. Germination rates were measured at the indicated number of days following imbibition.

All of the rice cultivars (wild-type, *s1*, *s2*, and *snat1+2*) had the same germination rates of approximately 90% after six days (Figure 5A). However, when assessed at three days, the degree of germination successes differed significantly between the wild-types and RNAi lines. The *s1* rice have three-day germination rates similar to that of the wild-types, whereas *s2* have three-day germination rates approximately half that of the wild-types. In contrast, *snat1+2* rice have three-day germination rates intermediate between the wild-types and *s1*. Consequently, the germination speed index was 2.0, 1.8, 1.1, and 1.3 for wild-type, *s1*, *s2*, and *snat1+2* rice, respectively (Figure 5B). These data suggest an antagonistic effect on germination speeds between *SNAT1* and *SNAT2*. Since germination and germination speeds are closely associated with GA, we measured the elongation patterns of third-leaf sheaths in response to GA_3_ treatments. The leaf sheath elongations assay is simple and more precise than the germination assay as a measure of GA involvement in certain physiological processes [27]. As shown in Figure 5C, treatments with GA_3_ (10 μM) caused a 1.5-fold increase in leaf sheath lengths of wild-type plants. Similarly, GA_3_ treatments of *s1*, *s2*, and *snat1+2* caused a similar 1.5-fold increase in leaf sheath lengths, although the total leaf sheath lengths were still shorter than those of the wild-types, indicating that the shorter leaf sheath lengths in the RNAi lines were not rescued by GA treatments. These data are consistent with a previous report that *s2* shoot lengths are insensitive to GA_3_ after pretreatments with the GA-inhibitor paclobutrazol [15].

### 3.5. Seed Viability

**Figure 6:**
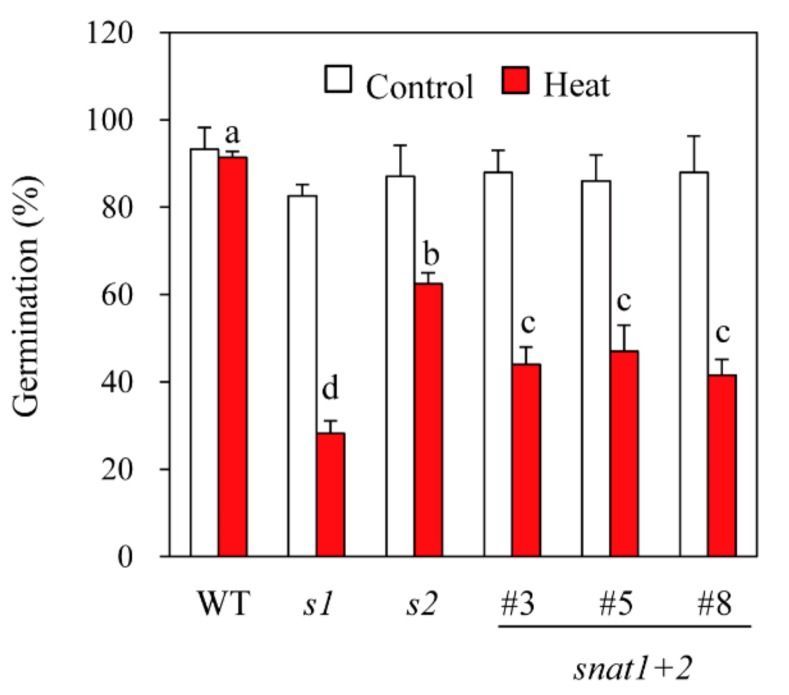
Seed germination in response to accelerated aging treatments. Rice seeds were exposed to heat (42 °C) under high humidity for four days, followed by germination at 28 °C for six days.

To test seed viability, rice seeds were exposed to a high temperature (42 °C) under 100% humidity for four days to accelerate seed aging [28]. After the accelerated aging treatment, seeds were allowed to germinate for six days at 28 °C. As shown in Figure 6, all of the genotypes (wild-types, *s1*, *s2*, and *snat1+2*) had 92% six-day germination rates in the absence of accelerated aging, but after accelerated aging, the germination rates declined to 24%, 62%, and 42% for *s1*, *s2*, and *snat1+2*, respectively. Interestingly, the germination rates of *snat1+2* after accelerated aging were intermediate between *s1* and *s2*, indicating that suppression of *SNAT2* is relatively protective of seed deterioration by accelerated aging. This observation suggests antagonistic physiological effects of *SNAT1* versus *SNAT2* in terms of seed viability.

## 4. Conclusions

Although the role of melatonin in plants is a relatively new field of study, compared with that in animals [2], much progress has been achieved since the first pharmacological study was reported in *Scadoxus multiforus* in 1969 [29]. The basic function of melatonin in plants is believed to act as a potent antioxidant, as it does in animals [4,30,31]. Thus, melatonin confers tolerance against various oxidative stresses caused by cadmium [32,33], herbicide [34,35], toxic compounds [9,36,37], and various abiotic stresses [38,39] in both plants and animals [2,40,41]. In addition to conferring tolerance against various abiotic stresses, melatonin is also implicated in several physiological and developmental processes, including maintenance of the endoplasmic reticulum [9]; sugar synthesis [42]; secondary metabolite synthesis [43,44]; growth [15]; somatic embryogenesis [45]; senescence [46,47]; root growth [48]; stomatal closure [8]; and flowering [49,50]. These diverse effects of melatonin are mediated by signaling molecules, such as H_2_O_2_ and NO, in combination with phytomelatonin receptors [6,8]. These data suggest that melatonin is multifunctional as both a hormone and biostimulator [1].

All genes responsible for melatonin biosynthesis in plants exist as a small gene family, except *T5H*. For example, *TDC* and *ASMT* exist in at least three copies in rice genomes [51,52], whereas *SNAT* has two copies with low amino acid homologies [53]. Among these four genes, *SNAT* seems to play an important role in synthesizing melatonin because it acts as a rate-limiting enzyme, and two *SNAT* isogenes play different physiological roles in rice plants. For example, *SNAT1* is positively associated with abiotic tolerance [12], whereas *SNAT2* is negatively associated with abiotic stress tolerance [15]. These different physiological roles of the *SNAT* isogenes may result from their interactions with BR. *SNAT1* functions independently of BR, whereas *SNAT2* functions in a manner that is dependent on BR [16].

Seeds lose viability during storage for a variety of reasons, including genetic damage, lipid peroxidation, and loss of membrane integrity [22]. Among these, lipid peroxidation is the major cause of seed deterioration. The relevance of melatonin to seed viability was first proposed by Manchester et al. [54], because melatonin is abundant in many edible seeds that are highly vulnerable to oxidative stress and storage. However, to date, no direct evidence has demonstrated that melatonin affects seed viability, although exogenous melatonin treatments do enhance germination rates in response to heat stress [55]. In this report, we have confirmed for the first time that endogenous melatonin levels are closely coupled with seed viability in rice. The largest decreases in germination rates after accelerated aging were observed in *snat1* rice, followed by *snat1+2* and *snat2*. The relatively mild suppression in *snat2* may be due to the simultaneous reduction of BR, which confers tolerance against various abiotic stresses [15]. Collectively, these results confirm and extend many previous reports that melatonin acts as a potent antioxidant that effectively prevents lipid peroxidation in living cells, as well as seeds in storage.
